# Supplementary material for: Machine learning enables completely automatic tuning of a quantum device faster than human experts
Source: Nat Commun. 2020 Aug 19;11:4161. doi: 10.1038/s41467-020-17835-9 (PMC7438325; doi:10.1038/s41467-020-17835-9)
Supplement: Supplementary file 1 — Supplementary Information [file 41467_2020_17835_MOESM1_ESM.pdf]

Machine learning enables completely automatic tuning of a quantum device faster than human experts

Moon, H., Lennon, D. T. et al.

SUPPLEMENTARY INFORMATION

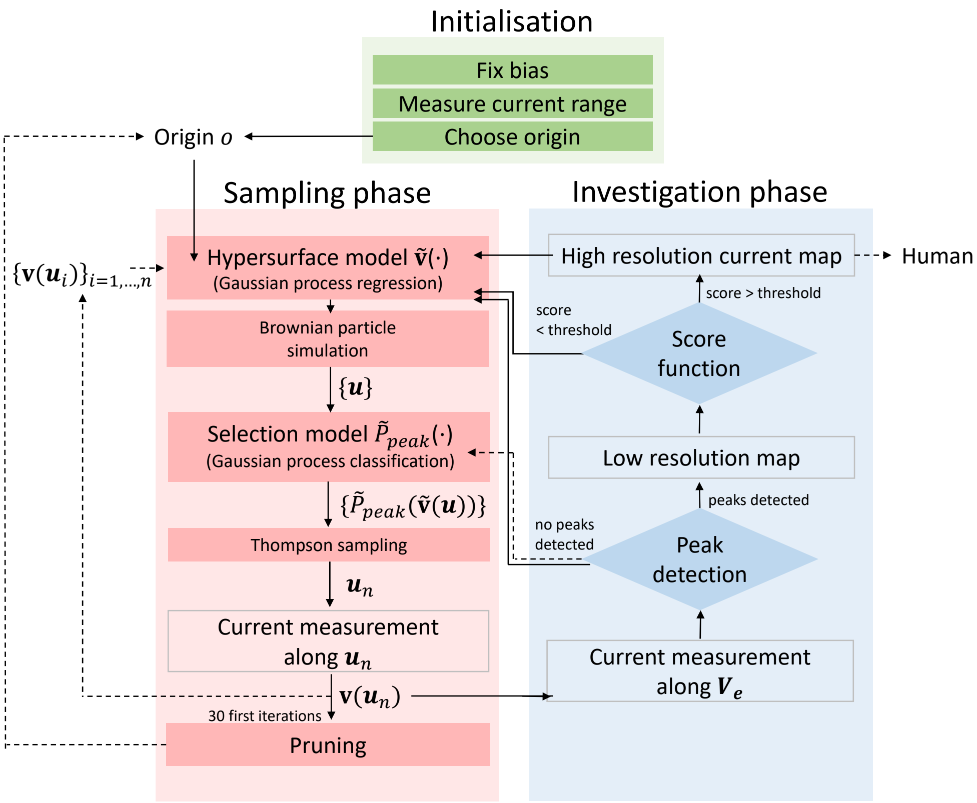

Supplementary Figure 1. **Detailed schematic of the entire algorithm.** Solid lines are control flows, and dotted lines are information flows. The most probable prediction of $\mathbf{v}(\mathbf{u})$ is given by $\tilde{\mathbf{v}}(\mathbf{u}) = m(\mathbf{u})\mathbf{u} + \mathbf{o}$. Other variables are defined in the main text.

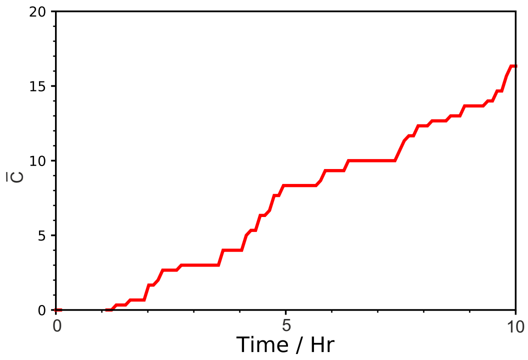

Supplementary Figure 2. **Grouped gates algorithm performance.** Average count of current maps displaying double quantum dot features, as a function of laboratory time for the grouped gates implementation of the algorithm. Current maps are labelled by humans a posteriori, i.e. after the algorithm is stopped.

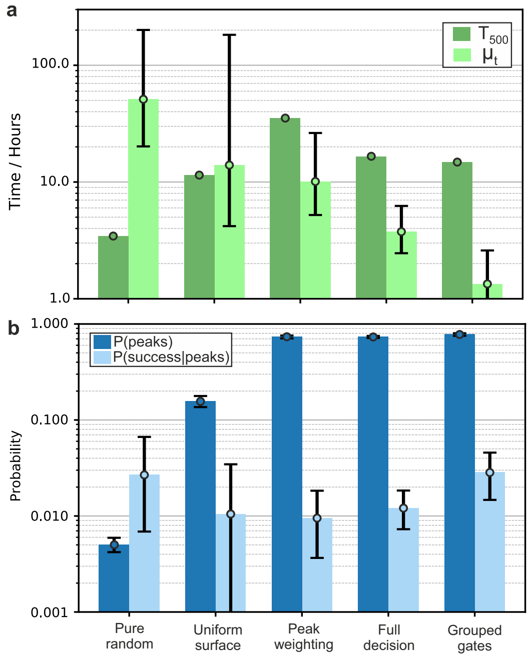

Supplementary Figure 3. **Additional ablation study data.** **a** and **b** bar charts comparing μ_t (light green), t_{500} (dark green), P(peaks) (dark blue) and P(success|peaks) (light blue) for the different algorithms considered in the ablation study with the inclusion of the grouped gates implementation. Error bars represent 10% and 90% confidence intervals.

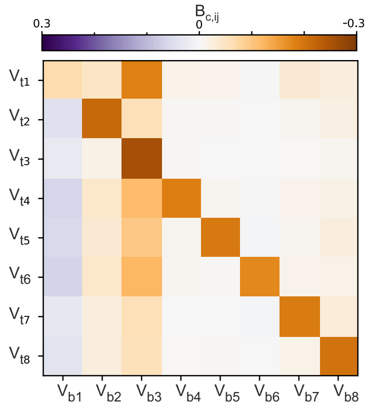

Supplementary Figure 4. **Additional device variability data.** The plot shows $B_c = B - I$ learned via point set registration from point sets $\mathbf{v}_\mathrm{a}$ and $\mathbf{v}_\mathrm{b}$ acquired on Device 1 before and after a thermal cycle.

Experiment	Iterations	Time (hours)	Labeller 1	Labeller 2	Labeller 3
Device1, run1	500	8.2	7	8	7
Device1, run2	500	9.1	7	8	6
Device1, run3	500	8.2	5	4	6
Device1, run4	500	8.4	5	11	10
Device1, run5	500	7.7	4	6	4
Device2, run1	500	11.2	2	3	1
Device2, run2	500	11.4	1	6	6
Device2, run3	500	11.2	0	4	2
Device2, run4	500	11.4	3	9	7
Device2, run5	500	10.5	3	4	5
Device2, Grouped gates, run1	500	11.1	12	19	20

Supplementary Table 1. Labelling result of the experiments in Section Device tuning of the main text. It shows the number of samples classified as successes for each labeller.

Experiment	Iterations	Time (hours)	Labeller 1	Labeller 2	Labeller 3
Pure random, run1	1000	6.9	0	0	0
Pure random, run2	10000	68.9	1	2	1
Uniform surface, run1	500	11.5	0	1	1
Peak weighting, run1	500	35.3	4	4	2
Full decision, run1	500	15.7	2	6	5
Full decision, run2	459	16.2	5	3	4
Full decision + grouped gates, run1	500	14.8	7	15	11

Supplementary Table 2. Labelling result of the experiments in Section Ablation study of the main text. It shows the number of samples classified as successes for each labeller. The second run of the Full method could not reach 500 iterations, because the algorithm was interrupted by some external noise.

SUPPLEMENTARY METHODS

Overview of the algorithm

We can motivate each component of the algorithm in a more conceptual way. Let $\mathcal{M}$ denote a R^{N-1} dimensional hyper-surface embedded in R^N. The algorithm is a random sampler on $\mathcal{M}$, which is optimised to decrease the expected tuning time (μ_t^{full} in Methods, Mathematical analysis of ablation study results). The algorithm achieves this by

- **(i)** running an approximately uniform random sampler on $\mathcal{M}$ (see main text sections Searching for the hypersurface, and Searching efficiently by learning the hypersurface),
- **(ii)** prioritising samples (see main text sections Searching for the hypersurface, Investigating nearby voltage space, and Searching efficiently by learning the hypersurface),
- **(iii)** rejecting unnecessary measurement (see main text section Investigating nearby voltage space).

We reformulate the expected tuning time for a simpler analysis:

$$\mu_t = \frac{\mu_i(c_{\text{peaks}}, c_{\text{2D-H}})}{P(\text{peaks})P(\text{success}|\text{peaks})}, \tag{1}$$

where $\mu_i(c_{\text{peaks}}, c_{\text{2D-H}}) = t_{\text{other}} + c_{\text{peaks}}(t_{\text{2D-L}} + c_{\text{2D-H}}\, t_{\text{2D-H}})$, which represents the expected time for each iteration. The denominator represents the probability of measuring double quantum dot transport features at each iteration. Note that an iteration means a run of the algorithm as indicated in Fig. 2b of the main manuscript. Equation (1) is an unified equation for μ_t^{full} and μ_t^{abl} in the manuscript. If the score function decision is disabled, then the algorithm always measures a high resolution current map, which makes $c_{\text{2D-H}} = 1$. If the score function decision is enabled, then the algorithm will measure a high resolution current map if the score function is higher than a threshold, which makes $c_{\text{2D-H}} = P(\text{highres}|\text{peaks})$. Likewise, the peak detection makes $c_{\text{peaks}} = P(\text{peaks})$, whereas it is 1 if disabled. It is trivial to see that μ_t^{full} and μ_t^{abl} are recovered when $c_{\text{2D-H}} = P(\text{highres}|\text{peaks})$ and $c_{\text{2D-H}} = 1$, respectively. The expected tuning time can be reduced by increasing the denominator or decreasing the numerator of μ_t. The purpose of **(ii)** is to increase the

denominator of μ_t, and the purpose of **(iii)** is to decrease the numerator of μ_t. Supplementary Figure 1 is a detailed block diagram of the algorithm.

Hypersurface detector

Finding the hypersurface boundary or 'pinch-off' boundary requires an expected maximum and minimum current value. This is obtained by a single current trace before running the algorithm. The pinch-off is defined by a threshold, chosen as 20% of the maximum current. The algorithm measures a trace in direction $\mathbf{u}$ from $\mathbf{o}$: $\mathbf{u}x_k + \mathbf{o}$, where k is the step number and x_k is the distance to the origin at the kth step. We choose $x_0 = 0$ and $x_{k+1} = x_k + 10$ mV. For each step k, the pinch-off detector identifies the gate voltage coordinate for which the measured current is lower than the threshold. If the current continues to be below the threshold for a certain range R, set to 50mV for all experiments, the algorithm considers the device is pinched off.

Gaussian process models

In this paper, we use two separate models that rely on Gaussian processes: one for estimating the hypersurface $r(\mathbf{u})$, and the other for estimating the location of Coulomb peaks $\tilde{P}_{\text{peak}}(\mathbf{v})$. Each Gaussian process requires a prior distribution, which is defined by a prior mean function and a covariance kernel. By setting the prior mean function $m_r(\cdot)$ and covariance kernel $k_r(\cdot,\cdot)$, the prior distribution is $\tilde{r}(\mathbf{u}) \sim \mathcal{N}(m_r(\mathbf{u}), k_r(\mathbf{u},\mathbf{u}))$, and the covariance is $\text{cov}[\tilde{r}(\mathbf{u}_1), \tilde{r}(\mathbf{u}_2)] = k_r(\mathbf{u}_1, \mathbf{u}_2)$.

For the model of $r(\mathbf{u})$ we initially have no prior knowledge, except for the maximum and minimum gate voltage bounds. Thus the range of r is: $r \in [r_{\min}, r_{\max}] = [0, \sqrt{N} \times 2\text{V}]$, where N is the number of gate electrodes. We set the prior distribution in this range: $m_r(\mathbf{u}) = (r_{\max} - r_{\min})/2$, $k_r(\mathbf{u},\mathbf{u}) = (r_{\max} - r_{\min})^2/4^2$. This prior means that $r_{\max}$ and $r_{\min}$ will be two standard deviations away from $m_r(\mathbf{u})$.

To model the probability $\tilde{P}_{\text{peak}}(\mathbf{v})$, we factorise it into two components $\tilde{P}_{\text{peak}}(\mathbf{v}) = \tilde{P}_{\text{peak}|\text{valid}}(\mathbf{v})\tilde{P}_{\text{valid}}(\mathbf{v})$, where $\tilde{P}_{\text{valid}}(\mathbf{v})$ is the probability that $\mathbf{v}$ corresponds to a pinch-off location, and not to the boundary of the operable gate voltages [0,-2] V. Each component of $\tilde{P}_{\text{peak}}(\mathbf{v})$ is estimated with a Gaussian process classification model.

For all Gaussian process models the Matern 5/2 kernel is used for the prior covariance. The prior covariance is defined by length scales l_k with $k = 1, ..., N$, setting the smoothness of the model predictions. These length scales are periodically optimised to the Maximum a posterior (MAP) using gathered data and a prior gamma distribution. The gamma distribution is set to have mean 0.4, and variance 0.1^2 for $r(\mathbf{u})$; mean 500 and variance 100^2 for $\tilde{P}_{\text{peak}|\text{valid}}$; and mean 50, and variance 20^2 for $\tilde{P}_{\text{valid}}$.

Score function

The score function is designed to distinguish between measurements containing double quantum dot features, single quantum dot features, and measurements for which the confinement potential is not well defined. This could be achieved using modern machine learning techniques, however, the score function we use is not reliant on training data and hence is less device and labeller specific. The score consists of three separate scores multiplied: the orientation of gradients score, the sharpness score, and the fit direction score.

The orientation of gradient score aims to capture if Coulomb peaks appear as straight lines in a current map or if a honeycomb pattern is present. This is achieved by taking the numerical derivative of the current map and producing gradient unit vectors. These gradient vectors will exhibit a distribution of angles. If Coulomb peaks appear as straight lines, then the distribution of angles will reveal two high-density collections of unit vectors, separated by π rad from each other. In this case, the high-density collections will have a small variance and a single line can fit the two points they define. For a honeycomb pattern, this variance will be larger or the high-density collections will not be well defined, and thus at least two lines will be required to fit the high-density collections. The value of the gradient orientation score is defined as the difference between the average of residuals corresponding to a fit through the high-density collections. Gradients smaller than a certain value are not considered for the fitting. This threshold was inferred from the current noise.

The sharpness score aims to prioritise 'sharp' Coulomb peaks. To compute the sharpness score the current map is first spit up into 16 smaller tiles. Each title is segmented into regions of high current and low current using a simple threshold. Then, the second order derivative of the current as a function of gate voltage is computed. This second order derivative is averaged over the high current regions and its standard deviation is calculated. The score for each tile is defined as the product of these average and standard deviation values. The scores for the 16 tiles are then averaged together giving the final sharpness score.

The fit direction score aims to further differentiate between single and double quantum dot behavior. The current map is first spit up into 16 smaller tiles, as for the sharpness score. For each of these tiles, a lines is fit to the high-density collections of gradient vectors, in the same way as for the orientation of gradient score. From this fit, a gradient vector angle is assigned to the tile. For each row of tiles (in direction $\hat{V}_e$), the standard deviation of gradient vector angles is calculated. In this way, the array of 4×4 tiles is reduced to a 1×4 vector. The average of the elements in this vector, defines the fit direction score.

Labelling procedure

Current maps that are measured by the algorithms in the tuning and ablation study sections, are labelled to determine if they contain features that indicate the device is tuned to the double quantum dot regime. This labelling is performed by human experts. To remove human bias, labelling was performed by 3 independent labellers.

Two separate data sets were labelled. The first contained 2048 current maps taken from all experiments presented in this paper, except the pure random experiment in Section Device tuning of the main text. The second contained 151 current maps, 51 of which were taken from the pure random experiment in the main text Section Device tuning and 100 were randomly selected from the first data set. The current maps in each data set were randomly shuffled so that labellers could not identify which experiment produced a given current map. The labellers were the same for both data sets.

Point set registration

Coherent point drift[1] was our choice of point set registration, since it can be used for point sets of high dimensionality. It is also simple to implement, it is robust to noise in the point sets, and supports affine transformations as well as rigid and non-rigid transformations. We focus on affine transformations $T(x) = Bx + t$, where B is a square matrix, and t is a column vector. Reference [1] presents the mathematical derivation for this case. In our application of the affine transformation, t does not have significance in terms of the device physics, and it was therefore set to 0. A derivation for the case $t = 0$ was required. This was achieved by setting the centered point matrices $\hat{X} = X$ and $\hat{Y} = Y$ in the derivation from section 4 (Rigid & affine point set registration) in Ref. [1].

Bayesian statistics

Single-labeller statistics

To infer the expected tuning time μ_t, and the probabilities $P(\text{peaks})$, and $P(\text{success}|\text{peaks})$, we use Bayesian inference with Jeffreys prior. Let p denote the probability (either $P(\text{peaks})$ or $P(\text{success}|\text{peaks})$) that we want to estimate. The Jeffreys prior for a binomial distribution is $p \sim \text{Beta}(0.5, 0.5)$. If we observe k successes over n trials, then the posterior distribution of p is $p|k, n \sim \text{Beta}(0.5 + k, 0.5 + n - k)$.

To infer μ_t, we assume that the rate of success over time follows a Poisson distribution $k \sim \text{Poisson}(\lambda t_{\text{tot}})$, where t_{tot} is the total time for an algorithm run, and λ is a rate parameter. The expected time between two consecutive successes (i.e. the acquisition of currents maps displaying double quantum dot transport features) is $\mu_t = 1/\lambda$. The Jeffreys prior for a Poisson distribution is $\lambda \sim \text{Gamma}(0.5, 0)$, hence the posterior is $\lambda|k, t_{\text{tot}} \sim \text{Gamma}(0.5 + k, t_{\text{tot}})$. Consequently, $\mu_t|k, t_{\text{tot}} \sim \text{Inv-Gamma}(0.5 + k, t_{\text{tot}})$.

Multi-labeller statistics

Before introducing multi-labeller statistics, let us introduce necessary notation: $\mathcal{D} = \{(\mathbf{v}_i, Y_i)|i = 1 \sim n\}$, where $\mathbf{v}_i$ is a voltage configuration, and Y_i is a high-resolution scan in the vicinity of $\mathbf{v}_i$. If no high-resolution scan was acquired at $\mathbf{v}_i$, then Y_i is an empty array. The data $\mathcal{D}$ requires a labelling function ψ: $\psi(Y_i) = 0$ if there is no recognized double quantum dot transport features, or 1 otherwise. But labellers might not agree, and thus we need to marginalise this effect. Let θ denote a parameter to be inferred (μ_t, P(peaks), or P(success|peaks)), then $p(\theta|\mathcal{D}, \psi) = p(\mathcal{D}|\theta, \psi)p(\theta)/p(\mathcal{D})$, where Ψ is a set of labellers. Note that ψ only affects the likelihood. To minimize the affect of ψ, the posterior can be marginalised over ψ and also approximated by samples of ψ:

$$p(\theta|\mathcal{D}) = \mathbb{E}_\psi[p(\theta|\mathcal{D}, \psi)] \approx \frac{1}{|\Psi|} \sum_{\psi \in \Psi} p(\theta|\mathcal{D}, \psi),$$

where $|\Psi|$ is the number of labellers. Therefore, the cumulative distribution function of $\theta|\mathcal{D}$ is

$$P(\theta < z|\mathcal{D}) \approx \frac{1}{|\Psi|} \sum_{\psi \in \Psi} P(\theta < z|\mathcal{D}, \psi).$$

Mathematical derivation of μ_t

We define t_i as the time taken to produce the ith sample, and $\bar{t}_i = \sum_{j=1}^{i} t_j$. We also define t_s as the interval between successes, or from start of the algorithm run to the first success if there were no previous success. Likewise, n_s is the iteration number corresponding to the first success. We want to derive $E[t_s]$. For brevity, we define shortened notations: $\alpha = P(\text{peaks})$, $\beta = P(\text{success}|\text{peaks})$. Note that $P(\text{success}) = \alpha\beta$.

Then the distribution of t_s is

$$P(t_s < t) = \sum_{i=1}^{n} P(n_s = i)P(\bar{t}_i < t|n_s = n).$$

The expected time is

$$\begin{aligned} E[t_s] &= \sum_{i=1}^{n} P(n_s = i)E[\bar{t}_i|n_s = i] \\ &= \sum_{i=1}^{n} (1 - \alpha\beta)^{i-1}\alpha\beta E[\bar{t}_i|n_s = i]. \end{aligned} \quad (2)$$

The last term $E[\bar{t}_i|n_s = i]$ depends on the tuning algorithm.

Derivation of μ_t^{abl}

The time for each iteration of the algorithm is $t_n = t_{n,\text{other}} + t_{n,\text{2D}}\mathbb{I}_{n,\text{peaks}}$, where $\mathbb{I}_{n,\text{peaks}}$ is equal to 1 if peaks are detected at $\mathbf{v}(\mathbf{u}_n)$ and 0 otherwise, $t_{n,\text{2D}}$ and $t_{n,\text{other}}$ is the time taken for 2D scans and other related times for iteration n. Consequently, $\bar{t}_n = \sum_{i=1}^{n} t_{i,\text{other}} + \sum_{i=1}^{n} t_{i,\text{2D}}\mathbb{I}_{i,\text{peaks}}$, and $\bar{t}_n|(n_s = n) = \sum_{i=1}^{n} t_{i,\text{other}} + \sum_{i=1}^{n-1} t_{i,\text{2D}}\mathbb{I}_{i,\text{peaks}} + t_{n,\text{2D}}$, because $n_s = n$ implies that 2D scans are measured at n. We assume that $t_{i,\text{other}}$ and $t_{i,\text{2D}}$ are i.i.d. across i, and we denote the expected time t_{others} and t_{2D}, respectively. Then the conditional expectation is

$$\begin{aligned}E[\bar{t}_n|n_s = n] &= nt_{\text{others}} + t_{\text{2D}} + (n-1)P(\text{peaks}|\text{fail at } n)t_{\text{2D}} \\ &= nt_{\text{others}} + t_{\text{2D}} + (n-1)\frac{(1-\beta)\alpha}{1-\alpha\beta}t_{\text{2D}},\end{aligned} \tag{3}$$

because $P(\text{peaks}|\text{fail at } n) = \frac{P(\text{fail at } n|\text{peaks})P(\text{peaks})}{P(\text{fail})} = \frac{(1-\beta)\alpha}{1-\alpha\beta}$. Substituting (3) into (2) results in

$$E[t_s] = \frac{t_{\text{others}}}{\alpha\beta} + \frac{t_{\text{2D}}}{\beta},$$

because of the following:

$$\begin{aligned}\alpha\beta\sum_{n=1}^{\infty}(1-\alpha\beta)^{n-1} &= 1 \\ \alpha\beta\sum_{n=1}^{\infty}(1-\alpha\beta)^{n-1}n &= \frac{1}{\alpha\beta} \\ \alpha\beta\sum_{n=1}^{\infty}(1-\alpha\beta)^{n-1}(n-1) &= \frac{1-\alpha\beta}{\alpha\beta}.\end{aligned}$$

Derivation of μ_t^{full}

For brevity, we use the following shortened notations: $\alpha' = P(\text{peaks}, \text{highres}) = P(\text{highres})$, $\beta' = P(\text{success}|\text{peaks}, \text{highres}) = P(\text{success}|\text{highres})$. Deriving the conditional expectation is very similar to the previous section,

$$\begin{aligned}E[\bar{t}_n|n_s = n] =& nt_{\text{others}} + t_{\text{2D-L}} + (n-1)\frac{(1-\beta)\alpha}{1-\alpha\beta}t_{\text{2D-L}} + \\ & t_{\text{2D-H}} + (n-1)\frac{(1-\beta')\alpha'}{1-\alpha'\beta'}t_{\text{2D-H}}.\end{aligned}$$

Therefore, the marginalised expectation is

$$E[t_s] = \frac{t_{\text{others}}}{\alpha\beta} + \frac{t_{\text{2D-L}}}{\beta} + \frac{t_{\text{2D-H}}}{\beta'}. \tag{4}$$

Note that $P(\text{success}) = \alpha\beta = \alpha'\beta'$, which leads to $\beta' = \alpha\beta/\alpha'$. Also, $\alpha/\alpha' = P(\text{peaks})/P(\text{highres}) = 1/P(\text{highres}|\text{peaks})$. Substituting these yields

$$E[t_s] = \frac{t_{\text{others}}}{\alpha\beta} + \frac{t_{\text{2D-L}}}{\beta} + \frac{P(\text{highres}|\text{peaks})t_{\text{2D-H}}}{\beta}.$$

Optimal threshold α'

An alternative expression for (4) is

$$E[t_s|\alpha', \beta'] = \frac{1}{\beta'}\left(\frac{t_{\text{OL}}}{\alpha'} + t_{\text{2D-H}}\right), \tag{5}$$

where $t_{\text{OL}} = t_{\text{others}} + \alpha t_{\text{2D-L}}$. Note that β' is the recall, which is the proportion of the real double dot samples over all positively classified samples, of a classifier, and it is related with α'. Therefore, a model of β' in terms of α' is required.

Perfect score function

We define a score function is perfect if there exists a threshold that perfectly classifies double-dot samples from others. In this case, The model for β' is

$$\beta' = \min(1, q/\alpha'),$$

where q is the actual proportion of double-dot samples. In other words, the recall is 1 if $\alpha' \leq q$, or q/α' otherwise. It is trivial to see that the expected time (5) is minimum at $\alpha' = q$. If q is unknown, which is usual, then we can utilise a prior distribution on q and optimise α' given the prior distribution.

In summary, from this analysis we conclude that it is beneficial to keep the threshold as big as possible if the recall does not drop significantly. Also, the incentive of using a score function as classifier is higher when the relative cost of a high resolution scan is big.

Imperfect score function

Let us assume that a score function is perfect with the probability ξ, or the score is random with the probability $(1-\xi)$. Then the model for β' is

$$\beta' = \frac{\min(q, \alpha')\xi + \alpha' q(1-\xi)}{\alpha'}.$$

Again, it is trivial that the expected time (5) increases when α' increases if $\alpha' \leq q$.

In case of $\alpha' > q$, (5) becomes

$$E[t_s|\alpha'] = \frac{1}{q}\frac{t_{\text{OL}} + \alpha' t_{\text{2D-H}}}{\alpha'(1-\xi) + \xi}.$$

and the partial derivative w.r.t. α' is

$$\frac{\partial E[t_s|\alpha']}{\partial \alpha'} = \frac{1}{q}\frac{t_{\text{2D-H}}\xi - (1-\xi)t_{\text{OL}}}{\{\alpha'(1-\xi) + \xi\}^2}.$$

Note that α' does not change the sign of the derivative, but ξ changes the sign. Rearranging $\frac{\partial E[t_s|\alpha']}{\partial \alpha'} = 0$ yields $\xi = \frac{t_{\text{OL}}}{t_{\text{OL}} + t_{\text{2D-H}}}$. It means that the optimal α' is q if $\xi > \frac{t_{\text{OL}}}{t_{\text{OL}} + t_{\text{2D-H}}}$. Otherwise, increasing α' always decreases the expected time, hence setting α' as big as possible reduces the time. It means that there is no advantage of using the score function if $\xi < \frac{t_{\text{OL}}}{t_{\text{OL}} + t_{\text{2D-H}}}$.

Additional results and raw data

Supplementary Figure 2 shows the tuning performance, on Device 1 (cool down 1), of the grouped gates implementation of the algorithm, under the same conditions as Section Device tuning in the main text. We have also included the grouped gates implementation of the algorithm, under the same conditions as in the main text Section Ablation study, in Supplementary Figure 3. Additional results for the device variability study in the main text Section Device variability, showing a thermal cycle of Device 1 (the thermal cycles compared are cool downs 1 and 2), are displayed in Supplementary Figure 4. The raw data used to calculate tuning performance in the main text Section Device tuning is shown in Supplementary Table 1. The raw data used to calculate tuning performance in the main text Section Ablation study is shown in Supplementary Table 2.

SUPPLEMENTARY REFERENCES

[1] Andriy Myronenko and Xubo Song, "Point set registration: Coherent point drifts," IEEE Transactions on Pattern Analysis and Machine Intelligence **32**, 2262–2275 (2010) .
