# Supplementary material for: Machine learning enables completely automatic tuning of a quantum device faster than human experts
Source: Nat Commun. 2020 Aug 19;11:4161. doi: 10.1038/s41467-020-17835-9 (PMC7438325; doi:10.1038/s41467-020-17835-9)
Supplement: Supplementary file 3 — Description of Additional Supplementary Files [file 41467_2020_17835_MOESM3_ESM.pdf]

Description of Additional Supplementary Files

File Name: Supplementary Data 1
Description: This file contains the original implementation of the algorithm. Detailed documentation can be found in "README.md" (markdown format, readable using an editor such as Typora or as plain text).

File Name: Supplementary Data 2
Description: This file contains a refactored implementation of the algorithm. This version contains example inputs, outputs, detailed documentation, and a mock environment so that the code can be tested without access to an experiment. Detailed documentation can be found in "README.html" and "Playground/README.html" (also available in markdown format, readable using an editor such as Typora or as plain text). We have also created multimedia files to help the readers understand our approach. A guided explanation of these animations can be found at AutoDot/Resources/Algorithm overview/README.html.
