# Supplementary material for: Machine learning enables completely automatic tuning of a quantum device faster than human experts
Source: Nat Commun. 2020 Aug 19;11:4161. doi: 10.1038/s41467-020-17835-9 (PMC7438325; doi:10.1038/s41467-020-17835-9)
Supplement: Supplementary file 5 — Supplementary Data 2 [file 41467_2020_17835_MOESM5_ESM.zip › AutoDot/Playground/README.html]

README


**running this demo with plot field set to true (like it is in the demo) imageio is required as an additional dependency.**

## How to run from scratch

If you're in a hurry do the following to run the demo:

**NOTE: DUE TO MULTIPROCESSING PACKAGE THE CURRENT IMPLEMENTATION ONLY WORKS ON UNIX/LINUX OPERATING SYSTEMS TO RUN ON WINDOWS FOLLOW THIS GUIDE**

```
```
conda create --name demo_test python=3.6
```


```
conda activate demo_test
```
```

To create and activate a new environment. If `conda activate demo_test` fails please try `source activate demo_test`.

```
```
xxxxxxxxxx
```


```
conda install scikit-image scipy numpy matplotlib
```


```
conda install -c conda-forge pydoe gpy
```
```

To install the required packages

Then in the parent folder of AutoDot:

```
```
xxxxxxxxxx
```


```
python
```
```

To open a shell

```
```
xxxxxxxxxx
```


```
from AutoDot.tune import tune_with_playground_from_file
```


```
tune_with_playground_from_file('AutoDot/mock_device_demo_config.json')
```
```

To run (if "plot":true you must close plots manually)

Outputs:
Plots (gifs) are saved in the folder 'mock\_device\_demo' and raw data it saved in a pickle file called 'tuning.pkl'. To open and read the pickle file run the following.

```
```
xxxxxxxxxx
```


```
import pickle
```


```
with open("mock_device_demo/tuning.pkl","rb") as handle:
```


```
    data_dict = pickle.load(handle)
```
```

data\_dict then contains all important output, for example `data_dict['vols_pinchoff']` contains a list of all observed pinch off values and boundary points.

If you're not in a hurry do the following sections might be helpful

## Before running

Provided already is a demo config json that will run 50 iterations of the algorithm as used in the paper with mock versions of the investigation stage steps in the 3D environment plotted below. The environment is specified to be 3D using the "ndim" field and the shape of the environment is specified using primitive shapes that are defined in shapes.py. The example used in the demo combines a standard Crosstalk\_box and Leakage shape (note leakage does not refer to leaky gates but refers to alterative undesired current pathways from source to drain). A 3D Crosstalk\_box is the type of shape one should expect to observe for three barrier gates. A Leakage demonstrates the pruning method and would be expected if one gate was required to define the current path (like a large top gate or V\_1 in the original paper).

The distribution of desired features can also be controlled by using the config. In the supplied demo the "investigation" field contains the following:

```
```
xxxxxxxxxx
```


```
"diag_trace":{
```


```
        "func":"mock_measurement",
```


```
        "condition":"mock_peak_check",
```


```
        "a":[0,0],
```


```
        "b":[-1000,-1000],
```


```
        "verbose":true},
```
```

Fields "a" and "b" define a box in gates 2 and 3 inside of which mock\_peak\_check will return true. For mock\_score\_func a Euclidian distance to "target" is used to define a score. Custom condition functions and measurement functions can be created by adding to condition\_functions.py and measurement\_functions.py. To see the configuration of a real experiments investigation stage see pygor\_demo\_config.json.

If the "plot" flag in "playground" is set to True the algorithm will first attempt to treat the environment as a 3D space and extract an isosurface (note this could not be done on a real device due to the time required to measure such a plot):

## During running

During running the algorithm will print many outputs. An example output for an iteration is:

```
```
xxxxxxxxxx
```


```
============### ITERATION 29 ###============
```


```
GPR: True GPC: True prune: True GPR1: True GPC1: True Optim: False
```


```
START
```


```
True
```


```
0.19766634805822517
```


```
Score thresh:  0.36156802062375365
```


```
STOP
```


```
dvec pinches:  [ True  True  True]
```


```
There are 30 training examples for model 0 and 26 are positive
```


```
There are 26 training examples for model 1 and 17 are positive
```


```
conditional_idx: 2
```


```
vols_pinchoff: [-1146.48524481  -813.5532      -647.01667969]
```


```
detected: True
```


```
r_vals: 1285.0125867654601
```
```

Below is a breakdown of what each line means:

```
```
xxxxxxxxxx
```


```
GPR: True GPC: True prune: True GPR1: True GPC1: True Optim: False
```
```

Denotes whether the gpr training, gpc training, gpr selection, gpc selection and gp optimisation is running for a given iteration

```
```
xxxxxxxxxx
```


```
START
```
```

Brownian motion sampling has started running in parallel with algorithm

```
```
xxxxxxxxxx
```


```
True
```
```

Result of mock\_peak\_check (only printed if "verbose":true)

```
```
xxxxxxxxxx
```


```
0.19766634805822517
```
```

Result of mock\_score\_func.

```
```
xxxxxxxxxx
```


```
Score thresh:  0.36156802062375365
```
```

Current value of the score threshold.

```
```
xxxxxxxxxx
```


```
STOP
```
```

Brownian motion sampling has stopped running in parallel with algorithm

```
```
xxxxxxxxxx
```


```
dvec pinches:  [ True  True  True]
```
```

If pruning is active this shows which gates are showing a pinch off

```
```
xxxxxxxxxx
```


```
There are 30 training examples for model 0 and 26 are positive
```


```
There are 26 training examples for model 1 and 17 are positive
```
```

Shows how many positives and training examples there are for the conditional gpc models (in the paper model 0 models if a pinch off will be observed and model 1 if a coulomb peak will be observed)

```
```
xxxxxxxxxx
```


```
conditional_idx: 2
```
```

Denotes how many stages in the investigation stage were passed. In the paper 0: no pinch, 1: no coulomb peak, 2: coulomb peak but poor regime, 3: nice regime. (NOTE only printed if "conditional\_idx" included in top level "verbose")

```
```
xxxxxxxxxx
```


```
vols_pinchoff: [-1146.48524481  -813.5532      -647.01667969]
```
```

Point of pinch off in voltage space. (NOTE only printed if "vols\_pinchoff" included in top level "verbose")

```
```
xxxxxxxxxx
```


```
detected: True
```
```

If pinch off was observed. (NOTE only printed if "detected" included in top level "verbose")

```
```
xxxxxxxxxx
```


```
r_vals: 1285.0125867654601
```
```

Distance from origin to the pinch off

## After running

All results are saved in the save directory ("save\_dir") in a file called tuning.pkl. The saved fields are specified in "track". Useful fields that you might want to look at are "vols\_pinchoff" (list of all pinch-off's or points where the algorithm hit the boundary), "detected" (if the point is a pinch-off or boundary point), "conditional\_idx" (denotes how successful a given iteration was at passing characterisation tests), "r\_vals" (distance from origin to the pinch-off/boundary), and "extra\_measure" (contains list of dicts that contain all information gathered by the investigation stage including stability diagrams if running on a real device).

If the "plot" flag in "playground" is set to True the algorithm will finally attempt to treat the fit Gaussian process prediction of the hypersurface (gpr) as a 3D space and extract an isosurface. It will also plot the Gaussian process classification (gpc) as the colour map of the gpr. The points used for training of these models will also be plotted:

Finally the improvement of the empirical probability of observing the situation that the gpc models is plotted as well:
