# Supplementary material for: Machine learning enables completely automatic tuning of a quantum device faster than human experts
Source: Nat Commun. 2020 Aug 19;11:4161. doi: 10.1038/s41467-020-17835-9 (PMC7438325; doi:10.1038/s41467-020-17835-9)
Supplement: Supplementary file 5 — Supplementary Data 2 [file 41467_2020_17835_MOESM5_ESM.zip › AutoDot/README.html]

README


# Quantum device tuning via hypersurface sampling

**NOTE: DUE TO MULTIPROCESSING PACKAGE THE CURRENT IMPLEMENTATION ONLY WORKS ON UNIX/LINUX OPERATING SYSTEMS TO RUN ON WINDOWS FOLLOW THIS GUIDE**

The quantum devices used to implement spin qubits in semiconductors can be challenging to tune and characterise. Often the best approaches to tuning such devices is manual tuning or a simple heuristic algorithm which is not flexible across devices. This repository contains the statistical tuning approach detailed in https://arxiv.org/abs/2001.02589 with some additional functionality, a quick animated explanation of the approach detailed in the paper is available here. This approach is promising as it make few assumptions about the device being tuned and hence can be applied to many systems without alteration. **For instructions on how to run a simple fake environment to see how the algorithm works see this README.html**

## Dependencies

The required packages required to run the algorithm are:

```
```
xxxxxxxxxx
```


```
scikit-image
```


```
scipy
```


```
numpy
```


```
matplotlib
```


```
GPy
```


```
mkl
```


```
pyDOE
```
```

# Using the algorithm

Using the algorithm varies depending on what measurement software you use in your lab or what you want to achieve. Specifically if your lab utilises pygor then you should call a different function to initiate the tuning. If you are unable to access a lab then you can still create a virtual environment to test the algorithm in using the Playground module. Below is documentation detailing how to run the algorithm for each of these situations.

## Without pygor

To use the algorithm without pygor you must create the following:

- jump
- measure
- check
- config\_file

Below is an **EXAMPLE** of how jump, check, and measure **COULD** be defined for a 5 gate device with 2 investigation (in this case plunger) gates.

jump:
Jump should be a function that takes an array of values and sets them to the device. It should also accept a flag that details whether the investigation gates (typically plunger gates) should be used.

```
```
xxxxxxxxxx
```


```
def jump(params,inv=False):
```


```
  if inv:
```


```
    labels = ["dac4","dac6"] #plunger gates
```


```
  else:
```


```
    labels = ["dac3","dac4","dac5","dac6","dac7"] #all gates
```


```
    
```


```
  assert len(params) == len(labels) #params needs to be the same length as labels
```


```
  for i in range(len(params)):
```


```
    set_value_to_dac(labels[i],params[i]) #function that takes dac key and value and sets dac to that value
```


```
  return params
```
```

measure:
measure should be a function that returns the measured current on the device.

```
```
xxxxxxxxxx
```


```
def measure():
```


```
  current = get_value_from_daq() #receive a single current measurement from the daq
```


```
  return current
```
```

check:
check should be a function that returns the state of all relevant dac channels.

```
```
xxxxxxxxxx
```


```
def check(inv=True):
```


```
  if inv:
```


```
    labels = ["dac4","dac6"] #plunger gates
```


```
  else:
```


```
    labels = ["dac3","dac4","dac5","dac6","dac7"] #all gates
```


```
  dac_state = [None]*len(labels)
```


```
  for i in range(len(labels)):
```


```
    dac_state[i] = get_current_dac_state(labels[i]) #function that takes dac key and returns state that channel is in
```


```
  return dac_state
```
```

config\_file:
config\_file should be a string that specifies the file path of a .json file containing a json object that specifies the desired settings the user wants to use for tuning. An example string would be "config.json". For information on what the config file should contain see the json config section.

### How to run

To run tuning without pygor once the above has been defined call the following:

```
```
xxxxxxxxxx
```


```
from AutoDot.tune import tune_from_file
```


```
tune_from_file(jump,measure,check,config_file)
```
```

## With pygor

To use the algorithm without pygor you must create the following:

config\_file:
config\_file should be a string that specifies the file path of a .json file containing a json object that specifies the desired settings the user wants to use for tuning. An example string would be "config.json". For information on what the config file should contain see the json config section. Additional fields are required to specify pygor location and setup.

### How to run

To run tuning with pygor once the above has been defined call the following:

```
```
xxxxxxxxxx
```


```
from AutoDot.tune import tune_with_pygor_from_file
```


```
tune_with_pygor_from_file(config_file)
```
```

## With playground (environment)

To use the algorithm using the playground you must create the following:

config\_file:
config\_file should be a string that specifies the file path of a .json file containing a json object that specifies the desired settings the user wants to use for tuning. An example string would be "config.json".

The config you must supply the field "playground" then in this field you must specify the basic shapes you want to build your environment out of. Provided is a demo config file and a README detailing how it works and what a typical run looks like.

### How to run

To run tuning with pygor once the above has been defined call the following:

```
```
from AutoDot.tune import tune_with_playground_from_file
```


```
tune_with_playground_from_file(config_file)
```
```
