# Supplementary material for: Machine learning enables completely automatic tuning of a quantum device faster than human experts
Source: Nat Commun. 2020 Aug 19;11:4161. doi: 10.1038/s41467-020-17835-9 (PMC7438325; doi:10.1038/s41467-020-17835-9)
Supplement: Supplementary file 5 — Supplementary Data 2 [file 41467_2020_17835_MOESM5_ESM.zip › AutoDot/Resources/Algorithm_overview/README.html]

README


# Algorithm overview

This file describes an animation of the algorithm that illustrates all its components. A '.mp4' animation containing 15 iterations of the algorithm (as detailed in our paper) in a 2D gate voltage space can be found here. Below we explain these iterations in detail.

## First few iterations

For the first few iterations (3 in this animation but 30 in the actual implementation), given an origin of the gate voltage space, candidate unit vectors are selected uniformly in angle. The pinch off is then found by moving to the origin and searching along the direction corresponding to the selected unit vector. This search ends if the current falls below a specific threshold or if the bounding box is hit (in this case -2 V for each gate voltage). When the pinch off is detected, a local search for Coulomb peaks is performed. The algorithm then starts pruning unpromising regions of the gate voltage space; this is done by moving closer to the origin in the direction of the unit vector (+100 mV in this example) and taking current traces parallel to the gate axes. During this procedure the pinch off locations (or if the search hit the bounding box) are logged.

### Iteration 1

In the example below a candidate unit vector is selected, and the algorithm performs a search along this direction. In this case, the search ends when the bounding box is hit, as no pinch off is found. A Coulomb peak check is performed, but no peaks are observed. The algorithm then starts pruning by first stepping back in the direction of the chosen unit vector and then performing two current traces parallel to the two axes of the voltage space. In this case, the first of these traces finds a pinch off location, but the second does not. This means that pruning is required to remove unpromising regions of gate voltage space from the search.

### Iteration 2

At the beginning of this iteration the origin is updated, following the results of the prunning performed in iteration 1. Another candidate unit vector is selected, and the algorithm performs a search along this direction from the updated origin. The search ends when a pinch off location is identified. A Coulomb peak check is performed, but again no peaks are observed. The algorithm continues with pruning by first stepping back in the direction of the chosen unit vector and then performing two current traces parallel to the two axes of the voltage space. Both of these traces find a pinch off location and thus no more prunning would be required.

## After the first few iterations

We will show 12 iterations of this stage (in a standard run of the algorithm approximately 470 iterations are required).

A Gaussian process regression model (hypersurface model) is used to predict the distance from the origin to the pinch off location (or bounding box) given a candidate unit vector. This model effectively aims to predict the hypersurface coordinates. A candidate unit vector is selected by simulating particles undergoing Brownian motion within the predicted hypersurface. Whenever one of these particles hits the hysurface, the corresponding location is added to a list of candidate locations.

A Gaussian process classification model is used to predict the probability of observing a Coulomb peak given a candidate location in gate voltage space. A candidate location is selected by sampling the list of candidate locations proportionally to the predicted probability of exhibiting Coulomb peaks in their vicinity (Thompson sampling). If all candidate locations are predicted to be equally likely to exhibit Coulomb peaks in their vicinity (this is always the case before the algorithm has performed enough measurements), then all candidate locations are equally likely to be selected.

After using the two models specified above to select a candidate location, the direction from the origin to this location is converted into a unit vector and the algorithm starts the search along this direction. To avoid returning to the origin for each iteration, the algorithm uses the hypersurface model to predict where the pinch off location might be. The search along the unit vector direction ends if the current falls below a specific threshold or if the bounding box is hit (in this case -2 V for each gate voltage). If the algorithm finds that the predicted pinch off location is already in the piched off region, the algorithm steps back in the direction of the unit vector towards the origin. When the pinch off location is detected, a local search for Coulomb peaks is performed.

### Iteration 4

The hypersurface model and Gaussian process classification model are used to choose a candidate unit vector. The algorithm uses the hypersurface model to predict the pinch off location, and its standard deviation to select a location to start the search. In this case, the predicted pinch off location is already in the piched off region, so the algorithm steps back towards the origin. Once the actual pinch off location is found, it is verified by moving along the unit vector in the opposite direction, i.e. away from the origin (this is too quick to appreciate in the animation). A Coulomb peak check is performed, and this time, they are observed (this is indicated in the animation by a green circle).

### Iteration 12

As both the hypersurface model and Gaussian process classification model have accumulated data from each iteration, they are able to make better predictions. This is reflected in measurements of the hypersurface being denser in regions exhibiting Coulomb peaks, and in predicted pinch off locations being very close in gate voltage space to observed pinch off locations.
