# Supplementary material for: Machine learning enables completely automatic tuning of a quantum device faster than human experts
Source: Nat Commun. 2020 Aug 19;11:4161. doi: 10.1038/s41467-020-17835-9 (PMC7438325; doi:10.1038/s41467-020-17835-9)
Supplement: Supplementary file 5 — Supplementary Data 2 [file 41467_2020_17835_MOESM5_ESM.zip › AutoDot/Resources/Running_on_windows.html]

Running\_on\_windows


# Running on windows

To run the algorithm on a windows computer you need to use a windows subsystem please follow the instructions at the following link: https://docs.microsoft.com/en-us/windows/wsl/install-win10.

We have tested Ubuntu 18.04 LTS and shown it works so it is preferred over other distributions.

# Setup subsystem

Once your windows subsystem is installed and you are logged-in and inside your ~ (home) directory run the following:

```
```
wget https://repo.continuum.io/miniconda/Miniconda3-latest-Linux-x86_64.sh
```
```

To download the latest version of miniconda.

```
```
xxxxxxxxxx
```


```
bash ./Miniconda3-latest-Linux-x86_64.sh
```
```

To install miniconda (please select default options).

```
```
xxxxxxxxxx
```


```
export PATH=~/miniconda3/bin:$PATH
```
```

To activate conda command.

# Install AutoDot

## From github (only available when repo is public)

```
```
xxxxxxxxxx
```


```
git clone https://github.com/oxquantum-repo/AutoDot.git
```
```

This will download a copy of the current repo. After this subsystem setup is complete and you can continue to follow either the quick environment guide or the main documentation.

## From zip

Download the AutoDot folder and unzip it into your windows downloads folder.

```
```
xxxxxxxxxx
```


```
cp -r /mnt/c/Users/<YOUR USERNAME HERE>/Downloads/AutoDot ~/AutoDot
```
```

Please replace <**YOUR USERNAME HERE**> with your windows username. This will copy and paste over the AutoDot folder in your downloads folder to the windows subsystem. After this subsystem setup is complete and you can continue to follow either the quick environment guide or the main documentation.
