# Supplementary figures and images for: Machine learning enables completely automatic tuning of a quantum device faster than human experts
Source: Nat Commun. 2020 Aug 19;11:4161. doi: 10.1038/s41467-020-17835-9 (PMC7438325; doi:10.1038/s41467-020-17835-9)

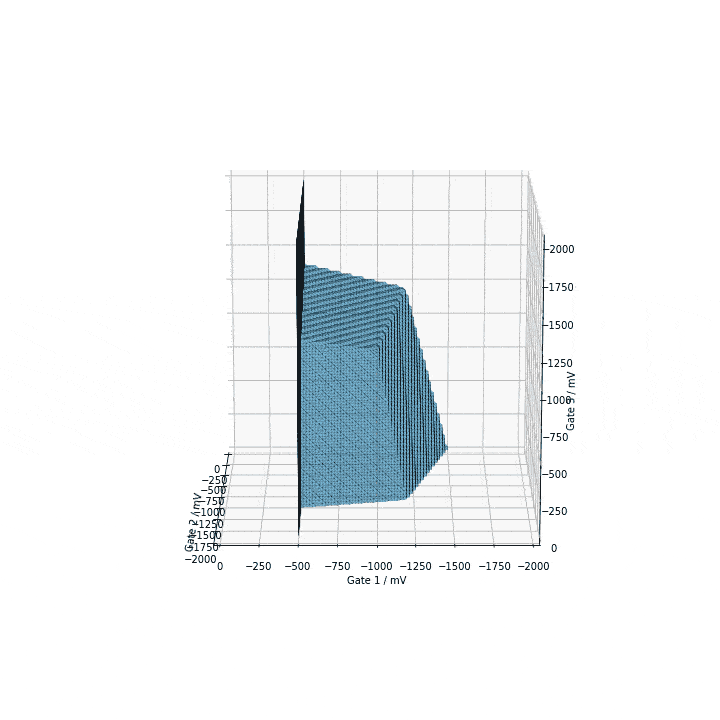

Supplement: Supplementary file 5 — Supplementary Data 2 [file 41467_2020_17835_MOESM5_ESM.zip › AutoDot/Playground/demo_run_data/color_comp_dummy.gif]

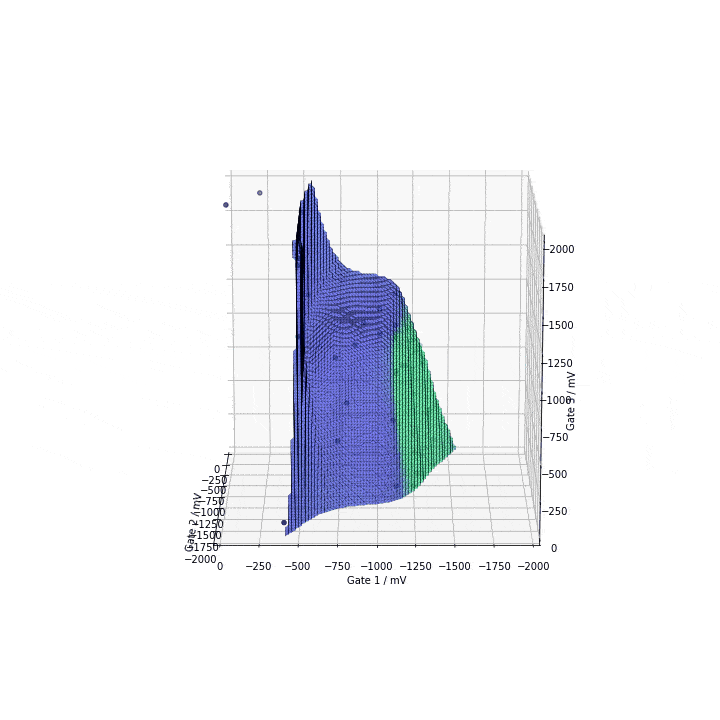

Supplement: Supplementary file 5 — Supplementary Data 2 [file 41467_2020_17835_MOESM5_ESM.zip › AutoDot/Playground/demo_run_data/gpr_and_gpc.gif]

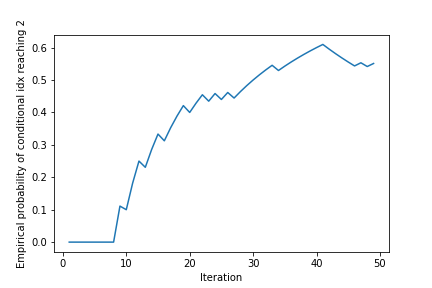

Supplement: Supplementary file 5 — Supplementary Data 2 [file 41467_2020_17835_MOESM5_ESM.zip › AutoDot/Playground/demo_run_data/improvment.png]

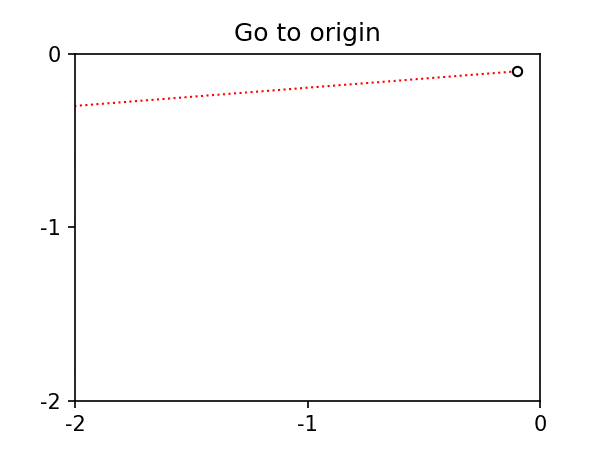

Supplement: Supplementary file 5 — Supplementary Data 2 [file 41467_2020_17835_MOESM5_ESM.zip › AutoDot/Resources/Algorithm_overview/iteration1.gif]

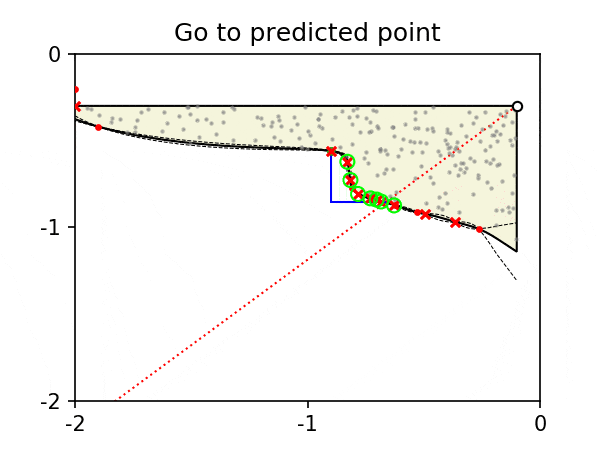

Supplement: Supplementary file 5 — Supplementary Data 2 [file 41467_2020_17835_MOESM5_ESM.zip › AutoDot/Resources/Algorithm_overview/iteration12.gif]

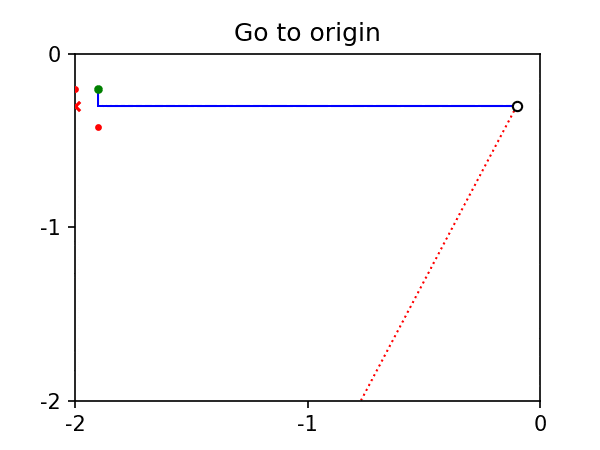

Supplement: Supplementary file 5 — Supplementary Data 2 [file 41467_2020_17835_MOESM5_ESM.zip › AutoDot/Resources/Algorithm_overview/iteration2.gif]

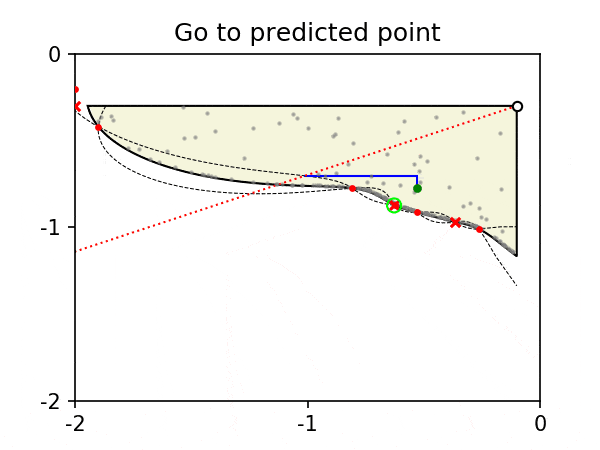

Supplement: Supplementary file 5 — Supplementary Data 2 [file 41467_2020_17835_MOESM5_ESM.zip › AutoDot/Resources/Algorithm_overview/iteration4.gif]
